# ORC6 acts as a biomarker and reflects poor outcome in clear cell renal cell carcinoma

**DOI:** 10.7150/jca.71313

**Published:** 2022-05-09

**Authors:** Qiufeng Pan, Fuchun Li, Yi Ding, Haoxuan Huang, Ju Guo

**Affiliations:** Department of Urology, The First Affiliated Hospital of Nanchang University, Nanchang, China.

**Keywords:** ORC6, origin recognition complex, renal cancer, biomarker, prognosis

## Abstract

**Purpose:** To explore the role of ORC6 in clear cell renal cell carcinoma (ccRCC).

**Methods:** The Cancer Genome Atlas Kidney Clear Cell Carcinoma (TCGA-KIRC) database was used to investigate the association between ORC6 expression and clinicopathological parameters. Furthermore, the expression level of ORC6 was determined in human RCC tissues and cell lines by western blot and PCR. Receiver operating characteristics curves and Kaplan-Meier curves were performed to assess the diagnostic and prognostic value of ORC6 in RCC.

**Results:** High expression of ORC6 predicted shorter overall survival (OS) (P<0.0001) and acted as an independent prognostic factor. ORC6 could distinguish the tumor from the normal patient (area under the curve=0.8827, P<0.0001). The expression of ORC6 was associated with the P53 signaling pathway, cell cycle, and DNA replication.

**Conclusion:** ORC6 could serve as a useful diagnostic and prognostic biomarker and a potential therapeutic target for ccRCC.

## Introduction

Clear cell renal cell carcinoma (ccRCC) accounts for ~80% of all diagnosed renal cell carcinoma (RCC), which is the most familiar malignancy of the adult kidney; in 2019, it is estimated that there are 14770 deaths and 73820 new cases of kidney malignancy 1, 2. Treatments of RCC vary a lot according to clinical staging; when the tumor is localized, patients could receive partial or radical nephrectomy; once the patients develop distant metastasis, chance of radical surgical operation is lost; timely diagnosis and treatment are crucial 3, 4. Although recent advances have been made in targeted therapies, patients still develop poor prognosis because of the secondary drug resistance 5, 6. Therefore, it is urgent and meaningful to investigate novel effective prognostic biomarker and elucidate underlying molecular mechanisms.

The origin recognition complex (ORC) is a highly conserved heterohexameric protein complex that associates with DNA at or near sites of initiation of DNA replication 7. All six ORC subunits are essential for initiation of DNA replication 8, and ORC may be involved in regulation of gene expression in response to stress 9. Modification of one or more of the six ORC subunits may be responsible for its inactivation during S phase 10. The roles of ORCs have been extensively investigated in both yeast and Drosophila 11, but information on human cancer is limited. To the best of our knowledge, the involvement of ORCs in ccRCC remains unknown.

In this study, we aimed to investigate the correlation of ORCs expression with clinicopathological parameters and patient survival in TCGA database. Experimental research in vitro revealed that knockdown ORC6 impaired malignancy of ccRCC. Our study demonstrated that ORC6 plays an oncogenic role and overexpression of ORC6 reflects poor prognosis. These findings showed that ORC6 may be a potential novel biomarker and target for better treatment in the future.

## 2. Materials and Methods

### 2.1. Patients samples

The Cancer Genome Atlas database of Kidney Clear Cell Carcinoma (TCGA-KIRC) comprises total 611 samples and 539 patients (https://tcga-data.nci.nih.gov/tcga/) 12. Surgical specimens of ccRCC patients who performed partial or radical nephrectomy were collected from the Department of Urology, the First Affiliated Hospital of Nanchang University between 2016 and 2020. Besides, the patients in the present study did not receive any adjuvant therapy before surgery and written informed consent was provided. The study methodologies conformed to the standards set by the Declaration of Helsinki and approved by the ethics committee of The First Affiliated Hospital of Nanchang University. Corresponding clinical pathological information of 50 pairs of patients was presented in Supplementary Table 1.

### 2.2. Bioinformatics analysis

A gene set enrichment analysis (GSEA) was conducted based on GSEA software (http://www.broadinstitute.org/gsea) 13. Nominal *P* value < 0.05 and a false discovery rate (FDR) < 25% were considered as statistical significant. Furthermore, an online STRING database was applied to retrieve protein-protein interaction network (https://string-db.org/) 14.

### 2.3. Cell culture and transient transfection

HK2, 786-O, Caki-1, ACHN, A-498, and OSRC-2 were purchased from The American Type Culture Collection (ATCC, USA). Cells were cultured in high glucose DMEM medium (Gibco; Thermo Fisher Scientific, Inc., Waltham, MA, USA) containing 10% fatal bovine serum (Gibco; Thermo Fisher Scientific, Inc., Waltham, MA, USA) and 1% streptomycin-penicillin in a 5% CO2 and 37°C incubator. As for transient transfection, small interfering (si) RNA oligonucleotide sequences specifically targeting ORC6 (siRNA) and negative control siRNA (Ctrl siRNA) were constructed by TranSheep Bio Co. Ltd. (Shanghai, China). The si-ORC6 sequence was as follows: siRNA-1, 5'-CCUUGGACAGGGUUAUUUTT-3'; siRNA-2, 5'-GGUUUGAACAAGGAGACAUTT-3'; siRNA-3, 5'-GCAGUGAACAUGGCUUCAATT-3'. Before transfection, According to the previous study^15^, cells were seeded in 6-well plates at 50%-70% confluence (3x10^5^) and were transfected with 100 pmol siRNA sequences using Lipofectamine® 2000 (Invitrogen; Thermo Fisher Scientific, Inc.). Then, cells were used for subsequent assays after 48h.

### 2.4. RNA extraction and RT-qPCR

Cell and tissue RNA was extracted using TRizol reagent (Thermo Fisher Scientific, Inc.). The concentration and purity of total RNA were detected by a NanoDrop 2000 spectrophotometer (NanoDrop Technologies; Thermo Fisher Scientific, Inc.). Then, total RNA was reverse transcribed to cDNA using a PrimeScript RT reagent Kit (Takara Bio, Inc.). RT-qPCR was performed according to the manufacturer's instructions (LightCycler 480II; Roche, Basel, Switzerland). Relative expression was calculated by the -2ΔΔCt method and GAPDH was used as an endogenous control. The PCR primers were chemically synthesized by TSINGKE.

GAPDH, 5'-AAAAGCATCACCCGGAGGAGAA-3' (forward) and 5'-AAGGAAATGAATGGGCAGCCG-3' (reverse); ORC6, 5'-ACCCCAAAGCACTGAGTTGA-3' (forward) and 5'-CGAACTCACCTCAGCATGTC-3' (reverse).

### 2.5. Western blotting

We followed the methods of Qiufeng Pan et al. 2020^16^. Cells and tissues were lysed in a protein lysis system including PMSF (Wuhan Boster Biological Technology, Ltd, Wuhan, China), protease inhibitor cocktail (Roche Diagnostics, Indianapolis, IN, USA), and RIPA buffer (Beyotime Institute of Biotechnology). Protein concentration was measured by the BCA kit (Beyotime Institute of Biotechnology). A total of 30μg proteins were presented and separated in 10% SDS-PAGE. Then, the gel system was transferred to polyvinylidene fluoride (PVDF) membranes (EMD Millipore, Bedford, MA, USA) for 90 mins at 90 V, after which the PVDF membrane was blocked in 5% nonfat milk dissolved in PBS for 1 h and then incubated with antibodies against ORC6 (1:1,000; A5426; Abclonal Biotec Co., Ltd) and β-actin (1:3,000; ab8226; Abcam Co., Ltd) at 4°C overnight. The next day, the membrane was washed and incubated with secondary antibodies (1:3,000; GB23303; Servicebio, Inc.) for 2 h at room temperature. Finally, the proteins were visualized with ChemiDoc-XRS^+^ (Bio-Rad Laboratories, Inc., Hercules, CA, USA).

### 2.6. Wound healing, transwell migration, and invasion assays

48h prior to the experiment, the 786-O cells was transfected with Ctrl-siRNA or siRNA. Cells were plated in 6-well culture plates in equal numbers. Vertical wounds were made with 10µl pipette tips, cell confluence reached approximately 95%, and the image were saved at 2, 12, and 24h. As for migration, and invasion assays, we also followed the methods of Qiufeng Pan et al 2020^16^. Cells were cultivated in DMEM without serum for 6-8 h to starve the cells before the migration and invasion assays. In this study, 24-well transwell chambers (Corning Incorporated, Corning, NY, USA) containing 8-μm membrane filters were used. Cells (1x10^4^) in 200 µl of serum-free medium were added into the upper chamber, whereas cells (2x10^4^) were inoculated into the upper chamber, which was pre-coated with Matrigel (BD, Franklin Lakes, USA) for invasion assay. Moreover, complete medium with 10% FBS was added to the bottom chamber. Incubation at 37˚C for 24 h, cells were fixed with 100% methanol for 10 mins, then stained with 0.05% crystal violet for 30 min at room temperature. Finally, five random fields were counted under a microscope (Olympus CX41-32C02; Olympus Corporation, Tokyo, Japan) at 100x magnification. Three independent experiments were conducted repeatedly.

### 2.7. Cell counting kit-8 (CCK-8) assay

Cells were added to the 96-well plate at a density of 1000 cells per well. Cell proliferation rate (OD value) was measured using Cell Counting Kit-8 reagent (CCK8, Djingo, Japan) at 24, 48, 72, 96 hours.

### 2.8. Statistical analysis

Statistical analysis was carried out by GraphPad Prism (version 7.0; GraphPad Software) and SPSS Statistics (version 22.0; IBM Corp.). Student's t-test was used to assess the difference in ORC6 expression between two groups; Pearson's χ^2^ test was used to evaluate the correlation between ORC6 expression and clinicopathological parameters of patients with ccRCC. The Kaplan-Meier (KM) curve evaluated patients' survival information and compared with Log-rank test; the Cox proportional hazard regression model was used to perform univariate and multivariate analysis. Data is shown as the mean ± SD. *P*<0.05 was regarded as significant.

## 3. Results

### 3.1. Relative expression of ORC family in ccRCC

**Figure 1:**
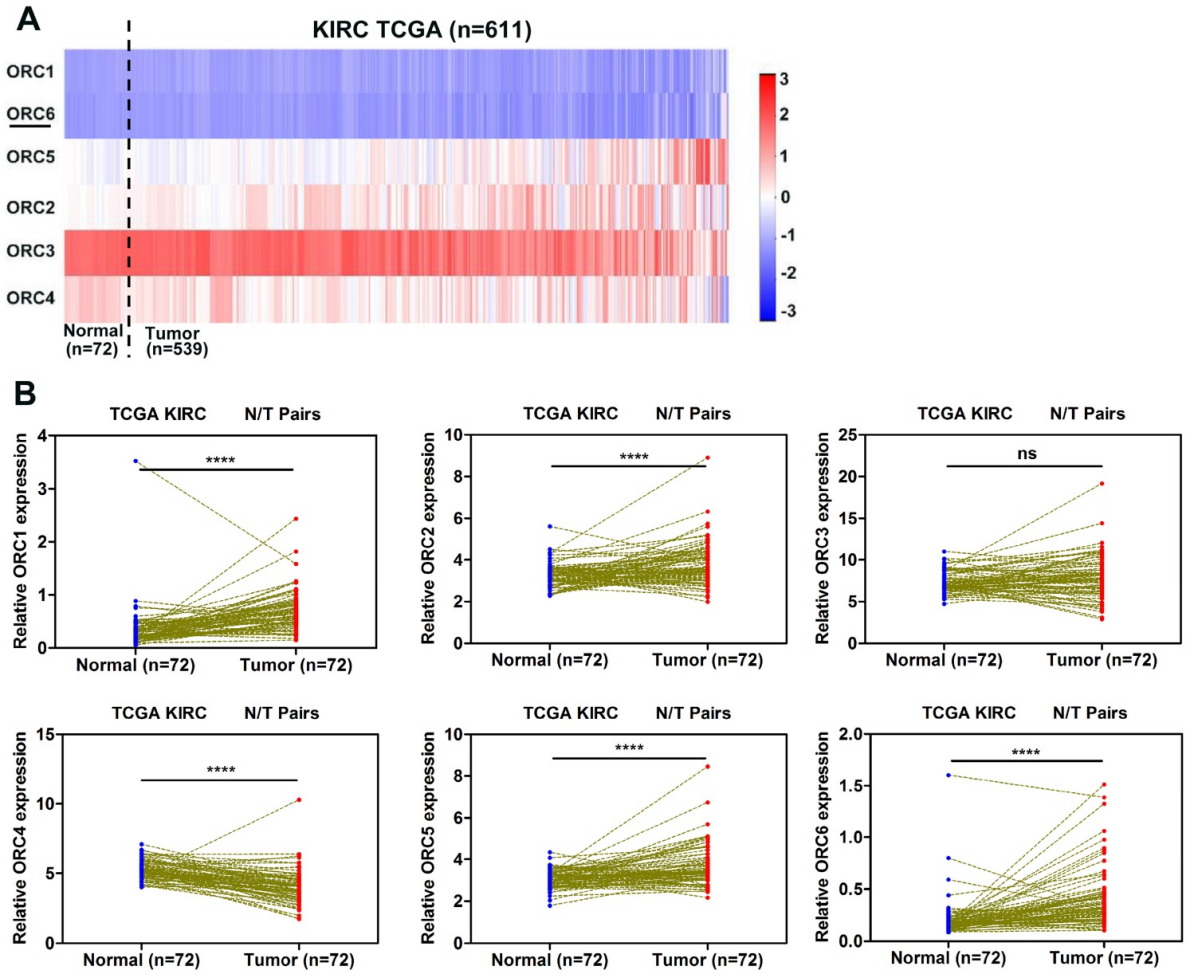
ORC family expression in TCGA-KIRC datasets. (A) Heat map revealing ORCs expression level in TCGA-KIRC datasets (n=611), (B) ORCs expression was compared in paired ccRCC tissues from the TCGA database. Red represents high expression; white represents medium expression; blue represents low expression. ORC, origin recognition complex; TCGA-KIRC, The Cancer Genome Atlas kidney renal clear cell carcinoma; ccRCC, clear cell renal cell carcinoma; N, normal; T, tumor; ****, *P*<0.0001; ***, *P*<0.001; **,* P*<0.01 and *, *P*<0.05; ns, no statistical significant.

To investigate the expression pattern of ORC family in ccRCC progression, we extractd the mRNA expression data of six ORC family members from the TCGA database. The heat map revealed the expression levels of ORC family members in Fig. 1A. Futhermore, the expression of each family member was analysed between ccRCC tissues and corresponding normal tissues. Compared with normal tissue, ORC1, ORC2, ORC5, and ORC6 exhibited significantly higher expression (Fig. 1B), ORC4 exhibited significantly lower expression in ccRCC and ORC3 showed no difference (Fig. 1B).

### 3.2. Prognostic and diagnostic significance of ORC family in ccRCC

**Figure 2:**
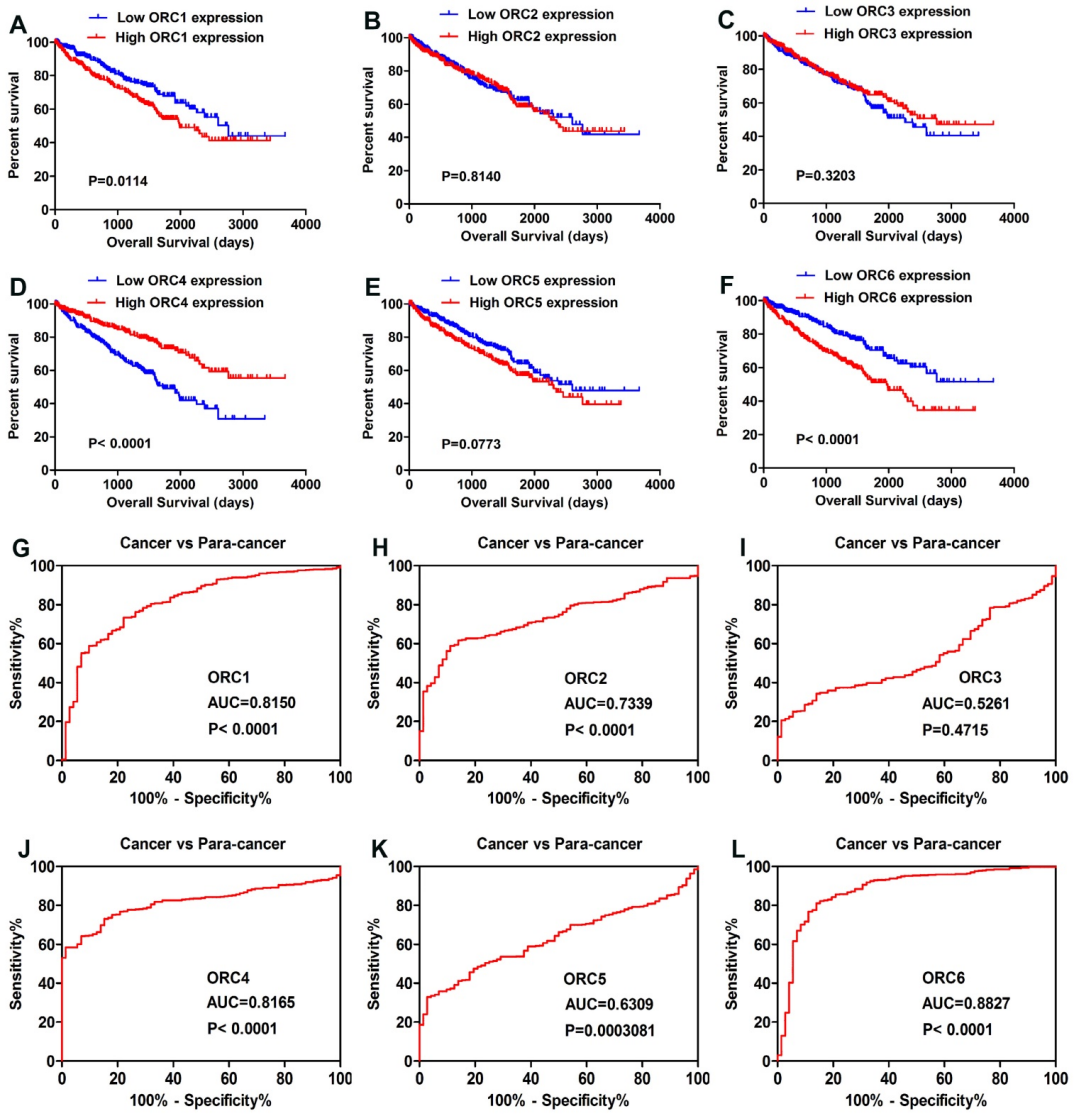
Prognostic and diagnostic role of ORC family. (A) High ORC1 expression had shorter OS, (B,C) Expression level of ORC2 and ORC3 had no effect on OS, (D) High ORC4 expression predicted favorable outcome, (E) Expression level of ORC5 showed no effect on OS, (F) High ORC6 expression reflected poor prognosis, (G,H) ORC1 and ORC2 effectively distinguished between ccRCC and paired normal tissues, (I) ORC3 failed to act as a diagnostic role, (J-L) ORC4, ORC5 and ORC6 exhibited diagnostic role. AUC, area under the curve; OS, overall survival; ORC, origin recognition complex.

All patients of TCGA-KIRC were divided into two groups according to the median expression level of the six ORC family members. Kaplan-Meier analysis revealed that higher expression of ORC1 and ORC6 predicted poorer overall survival (OS) (Fig. 2A, F), and that higher ORC4 indicated better OS (Fig. 2D). However, no difference in OS was found with regard to ORC2, ORC3, and ORC5 (Fig. 2B,C,E). Futhermore, we performed ROC curve analysis of ORC family members to investigate its diagnostic role. The results showed that ORC1 (Fig. 2G, AUC=0.8150, P<0.0001), ORC2 (Fig. 2H, AUC=0.7339, P<0.0001), ORC4 (Fig. 2J, AUC=0.8165, P<0.0001), ORC5 (Fig. 2K, AUC=0.6309, P=0.0003081), and ORC6 (Fig. 2L, AUC=0.88270, P<0.0001) could effectively distinguish ccRCC patients. Unfortunately, ORC3 failed to act as a diagnostic factor (Fig. 2I, AUC=0.5261, P=0.4715).

### 3.3. Multivariate analysis of overall survival (OS) in ccRCC

To further evaluate the prognostic value of ORC family, we selected the expression levels of ORC1 and ORC6 (Low versus High), age, gender, pathological grade, T stage, N stage, M stage, TNM stage to construct multivariate analysis of OS. Multivariate analysis demonstrated that ORC6 could be treated independent prognostic factor (HR=1.414, P=0.039, Table 1). However, ORC1 failed to be an independent prognostic factor (P=0.463, Supplementary Table 2). Therefore, we focused on ORC6 and demonstrated how ORC6 participated in ccRCC development.

### 3.4. ORC6 expression level predicted unfavorable clinical outcome

**Figure 3:**
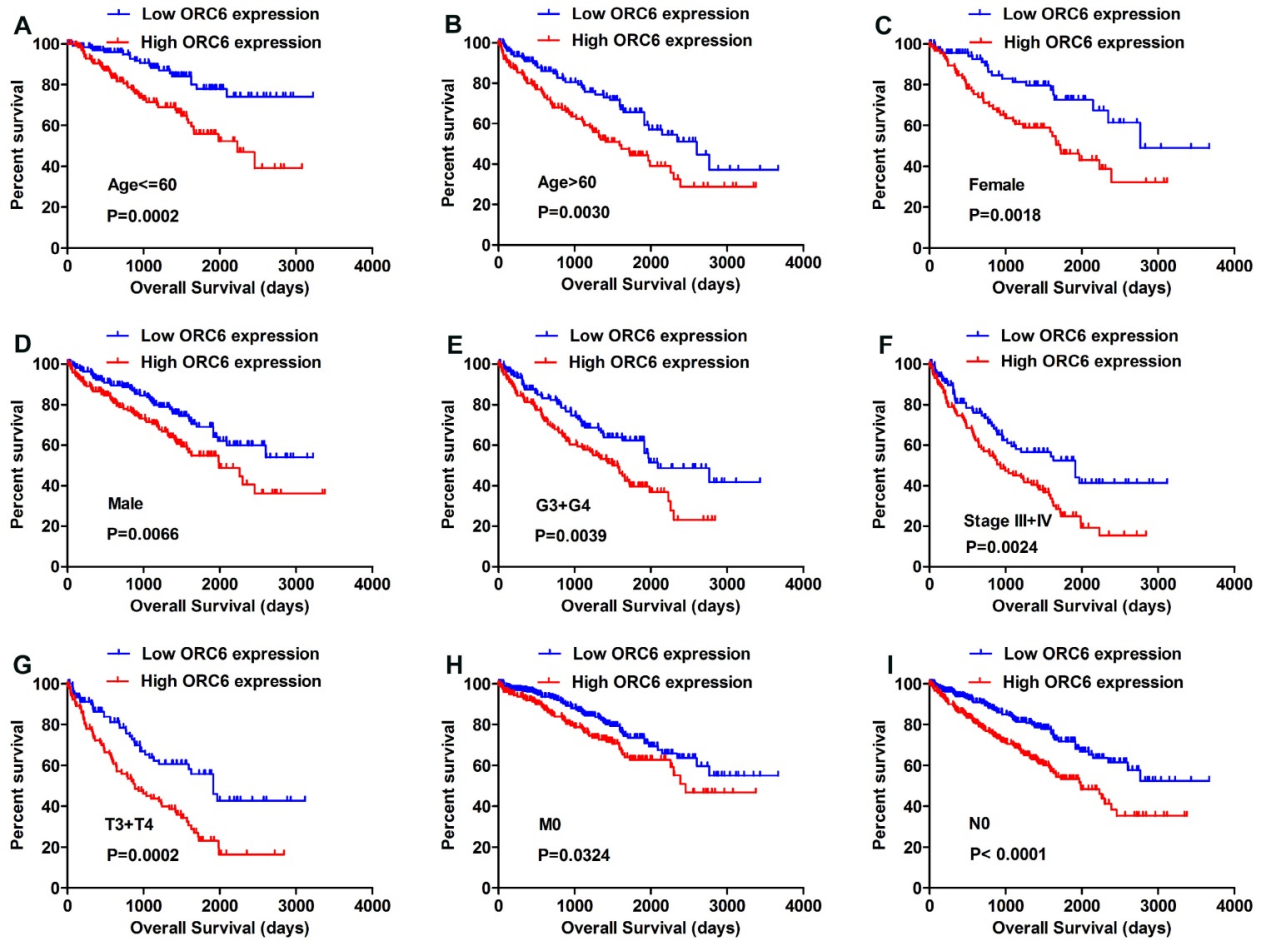
Correlation of ORC6 expression and overall survival (OS) in TCGA-KIRC subgroup patients. (A) age<=60, (B) age>60, (C) Female, (D) Male, (E) G3+G4, (F) Stage III+IV, (G) T3+T4, (H) M0, (I) N0. ORC, origin recognition complex; TCGA-KIRC, The Cancer Genome Atlas kidney renal clear cell carcinoma.

We applied the Kaplan-Meier survival analysis to determine OS in different subgroups of patients according to the median ORC6 expression level. The results demonstrated that ORC6 may be a potential prognostic indicator for patients with following clinical features: Age <=60 (Fig. 3A) or Age >60 (Fig. 3B), Female (Fig. 3C) or Male (Fig. 3D), G3+G4 (Fig. 3E), Stage III+IV (Fig. 3F), T3+T4 (Fig. 3G), M0 stage (Fig. 3H), and N0 stage (Fig. 3I).

### 3.5. ORC6 served as a diagnostic factor for ccRCC subgroup patients

**Figure 4:**
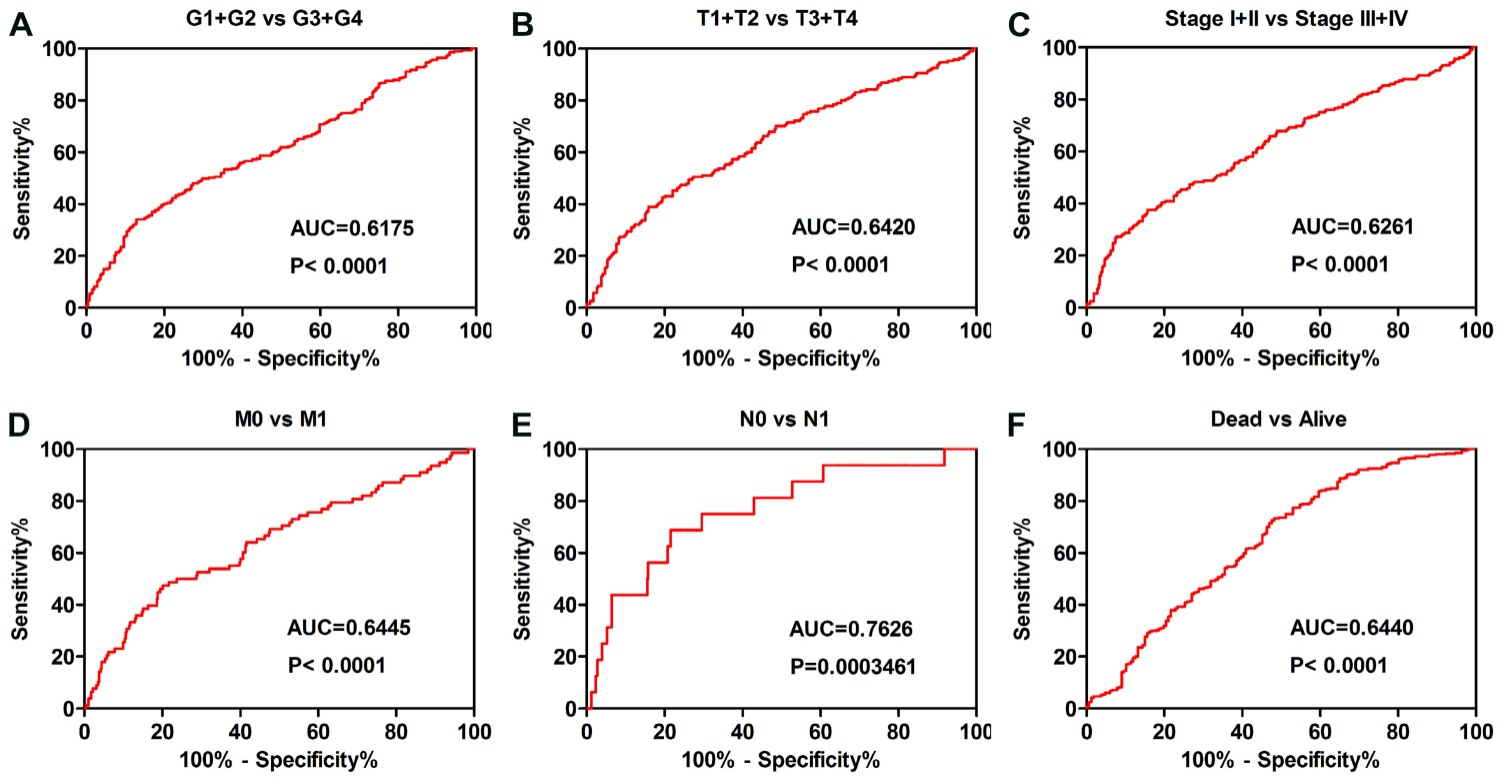
ROC curve analysis was conducted in subgroup of patients with ccRCC. (A) grade, (B) T stage, (C) TNM stage, (D) Distant metastasis, (E) Lymph node metastasis, (F) vital status. ccRCC, clear cell renal cell carcinoma; AUC, area under the curve; ROC, receiver operating characteristic.

The ORC6 expression level also played diagnostic role for patients as follows: G1+G2 vs G3+G4 grade (Fig. 4A AUC=0.6175 P<0.0001), T1+T2 vs T3+T4 stage (Fig. 4B AUC=0.6420 P<0.0001), Stage I+II vs Stage III+IV (Fig. 4C AUC=0.6261 P<0.0001), M0 vs M1 stage (Fig. 4D AUC=0.6445 P<0.0001), N0 vs N1 stage (Fig. 4E AUC=0.7626 P=0.0003461), Alive vs Dead (Fig. 4F AUC=0.6440 P<0.0001).

### 3.6. ORC6 expression level promoted ccRCC progression

**Figure 5:**
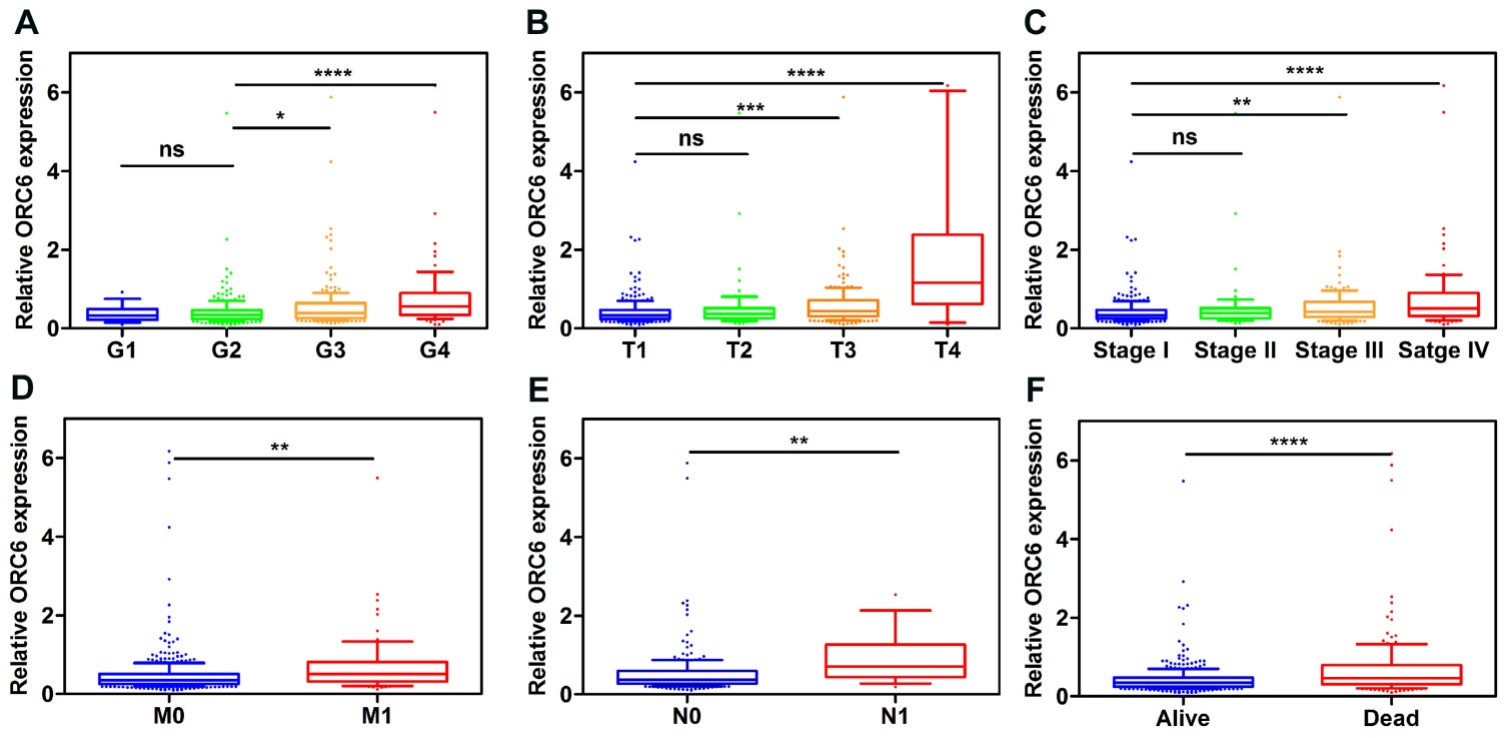
ORC6 expression level is closely correlated with various clinicopathological parameters. (A) Grade, (B) T stage, (C) TNM stage, (D) Distant metastasis, (E) Lymph node metastasis, (F) Vital status. ccRCC, clear cell renal cell carcinoma; ****, *P*<0.0001; ***, *P*<0.001; **,* P*<0.01 and *, *P*<0.05. ns, no statistical significant.

Patients with complete clinical information in TCGA-KIRC (n=530) were separated according to ORC6 median expression level. Pearson's χ^2^ test demonstrated that the expression level of ORC6 were associated with patients' age, histological grade, TNM stage, T stage, N stage, M stage and Survival status (Table 2). Furthermore, higher expression levels of ORC6 were associated with higher pathological grade, TNM stage, higher tumor T stage, distant metastasis, lymph node metastasis in ccRCC (Fig. 5A-F).

### 3.7. Upregulation of ORC6 was further verified in ccRCC cells and tissues

**Figure 6:**
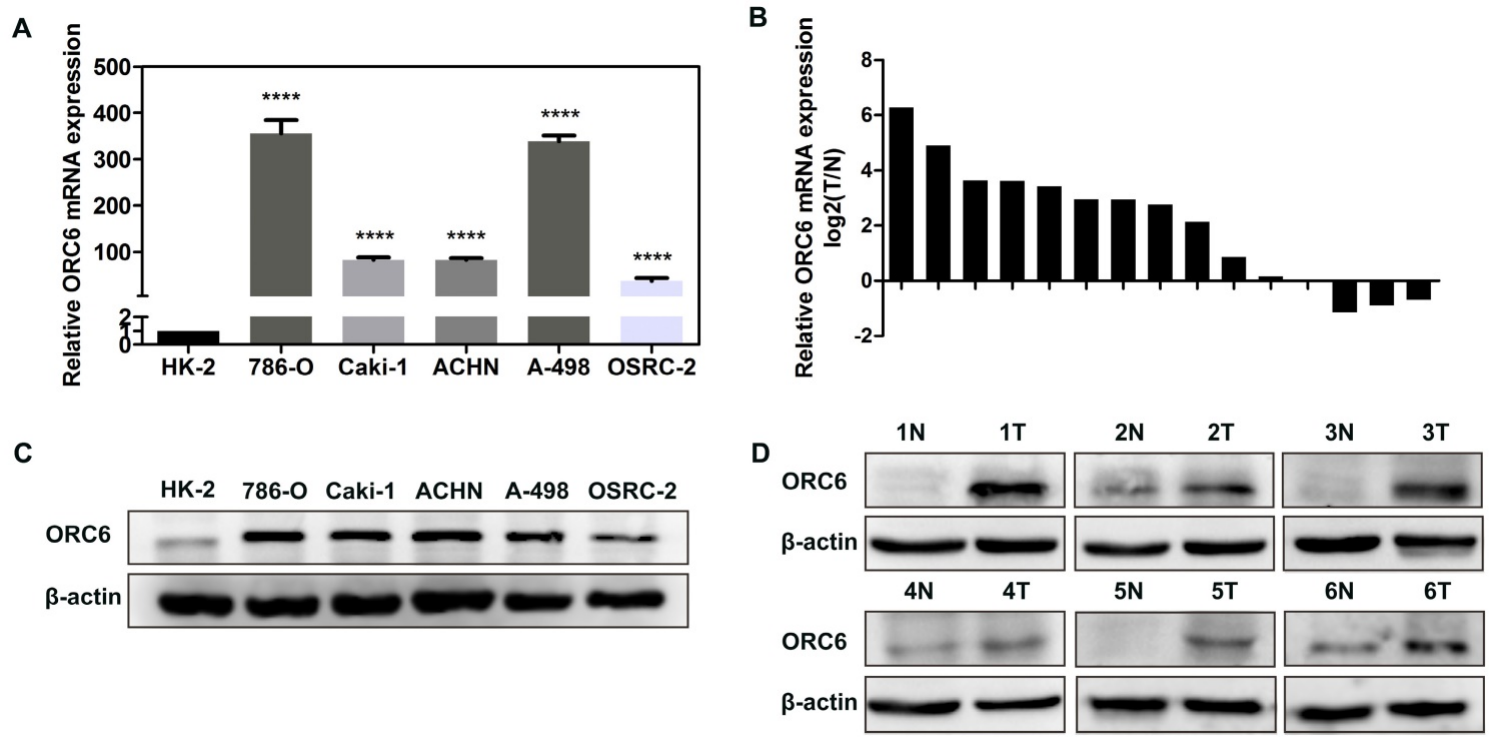
ORC6 is upregulated in cancer cells and ccRCC tissues. (A) and (B) qRT-PCR analysis of ORC6 mRNA expression in cancer cells and ccRCC tissues, (C) and (D) Western blot assay analysis of ORC6 protein expression in cancer cells and ccRCC tissues. qRT-PCR, quantitative real-time polymerase chain reaction; ccRCC, clear cell renal cell carcinoma; N, normal; T, tumor; ****, *P*<0.0001; ***, *P*<0.001; **,* P*<0.01 and *, *P*<0.05.

We performed RT-qPCR and western blot assays to validate the results from public database. RT-qPCR assay indicated that ORC6's mRNA expression in ccRCC cells (786-O, Caki-1, ACHN, A-498, OSRC-2) was significantly higher than HK-2 cells (Fig. 6A), and that compared with normal tissues, ORC6's mRNA expression in ccRCC tumor tissues was also higher (13 cases of 15 cases were upregulated, Fig. 6B). Moreover, western blot assay demonstrated that ORC6's protein level was upregulated in ccRCC cells and tissues (Fig. 6C,D).

### 3.8. Pathway and biological pathogenesis of ORC6 in ccRCC

**Figure 7:**
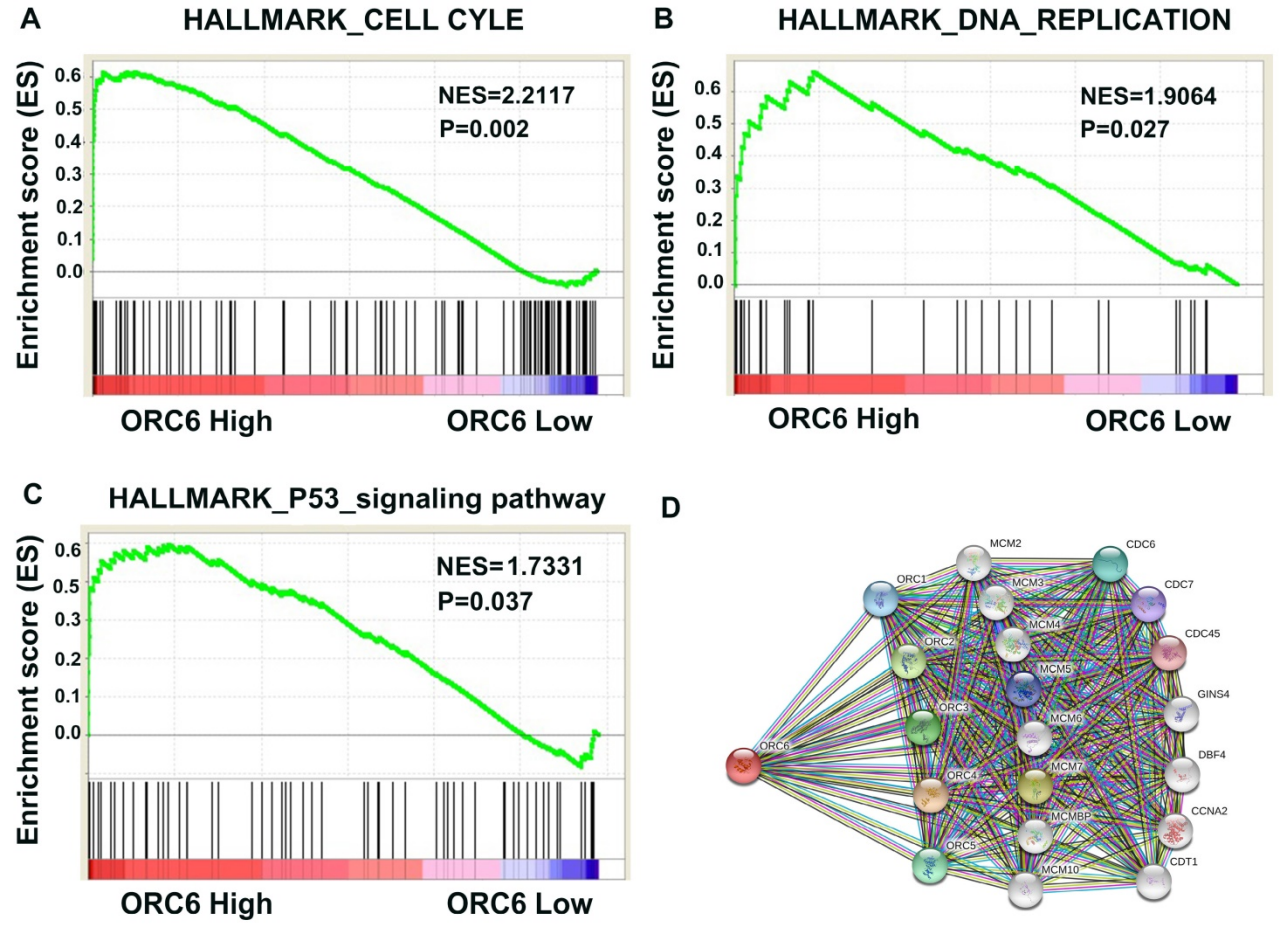
Pathway involved in the pathogenesis of ORC6 in TCGA-KIRC with GSEA and PPI. (A) Cell cycle, (B) DNA replication, (C) P53_signaling pathway, (D) Protein-protein interaction network. KEGG, Kyoto Encyclopedia of Genes and Genomes; GSEA, gene set enrichment analysis; PPI, protein-protein interaction network.

Based on above results, we were eager to know how ORC6 drived ccRCC progression. Then we performed GSEA analysis in TCGA database and retrieved the “STRING” database. The results revealed that ORC6 expression was correlated with gene signatures of cell cycle, DNA replication, and P53 signaling pathway (Fig. 7A-C). Moreover, many proteins interacted with ORC6 including ORC family, MCM family, and CDC family (Fig. 7D).

### 3.9. Knockdown of ORC6 impaired malignancy of ccRCC in vitro

**Figure 8:**
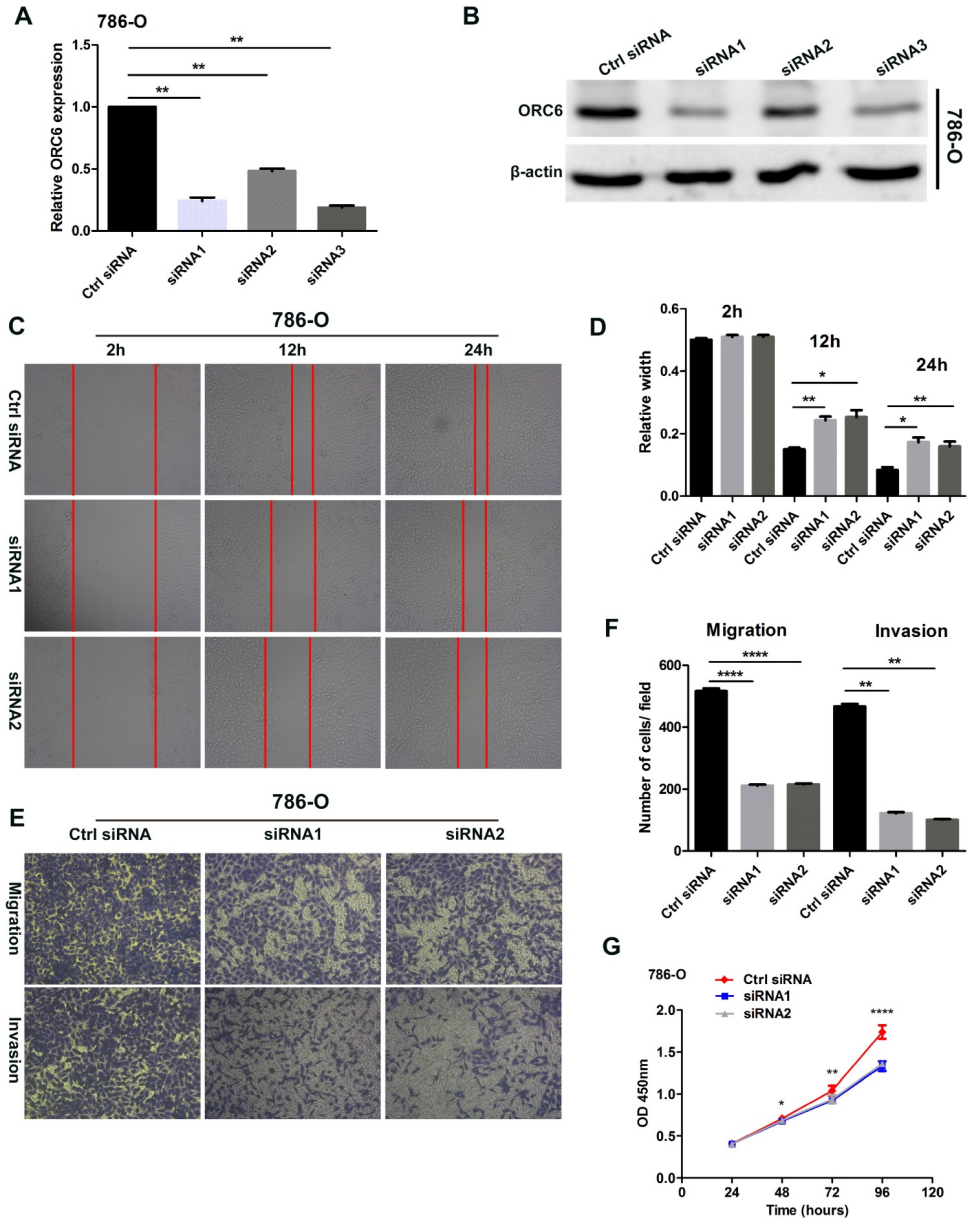
Downregulation of ORC6 impaired malignancy of ccRCC in vitro. (A) and (B) Knockdown of ORC6 expression in 786-O cells with siRNA (siRNA1, siRNA2 and siRNA3), (C) and (D) Silence ORC6 significantly suppress the scratch healing capacity of 786-O cells, (E) and (F) Knockdown of ORC6 repressed the migration and invasion of 786-O cells, (G) Silence ORC6 significantly repressed the proliferation of 786-O cells. The wound healing assays were imaged at 2, 12, and 24h after scratches were made; Each experiment was performed at least three times and data was shown as mean ± SD; original magnification was x100; siRNA, small interfering RNA; ****, *P*<0.0001; ***, *P*<0.001; **,* P*<0.01 and *, *P*<0.05; ns, no statistical significant.

Three ORC6 siRNAs were transfected into 786-O cells, RT-qPCR and western blot assays demonstrated that three siRNAs successfully knock down ORC6 in 786-O cells (Fig. 8A,B). Wound healing assays indicated that the knock down of ORC6 could inhibit the scratch healing ability (Fig. 8C, D). In transwell assays, 786-O normal control cells exhibited greater cell migration and invasion ability than knockdown cells (Fig. 8 E, F). Moreover, CCK8 assays revealed that cell proliferation was markedly attenuated in knockdown cells (Fig. 8G). Collectively, downregulation of ORC6 suppressed malignancy of ccRCC in vitro.

## 4. Discussion

Clear cell renal cell carcinoma (ccRCC) is a highly heterogeneous disease, exhibiting high invasiveness and metastasis propensity 17. Exploring novel effective biomarker and molecular mechanism in tumor progression may contribute to assessing and managing patients. The origin recognition complex (ORC), a six-protein complex, binds replication origin DNA, recruits other initiation factors and facilitates loading of the DNA helicase 7. ORC6, however, appears no structural similarity to the other ORC proteins, and is the only ORC subunit not required for DNA binding 18, 19. Nevertheless, to our knowledge, little is known about how ORC6 is involved in ccRCC pathogenesis.

In this study, we investigated expression of six ORCs family members and patient survival in TCGA database. We found that: (I) expression of ORC1, ORC2, ORC5, ORC6 are upregulated, while ORC4 is downregulated in tumor; (II) high expression of ORC1 and ORC6 predicted poor prognosis in ccRCC, while ORC4 predicted favorable prognosis; (III) the ORC6 expression level was an independent prognostic factor in TCGA-KIRC; (IV) high ORC6 expression was closely correlated with grade, T stage, TNM stage, N stage, M stage, and vital status; (V) high ORC6 expression was associated with the gene set of P53 signaling pathway, cell cycle, and DNA replication; (VI) knockdown ORC6 repressed malignancy of ccRCC in vitro.

Supriya G Prasanth et al. implicated that silencing of ORC6 caused a decrease in cell proliferation and increased cell death, and that ORC6 as an essential gene that coordinated chromosome replication and segregation with cytokinesis 20. Shuyan Chen et al. demonstrated that lacking ORC6 inhibits the interacting with Cdt1 and loading the Mcm2-7 helicase onto origin DNA, thus hindering the DNA replication initiation 21. Furthermore, in human colon cancer cells, Etsuko Shibata et al. reported that cells lacking detectable ORC1, ORC2 or ORC5 still grow, recruit MCM2-7 and initiates DNA replication; this implied that ORC1 or ORC2 or ORC5 seems dispensable to DNA replication 22. In pancreatic cancer, Polo-like kinase 1 phosphorylation of ORC2 maintains DNA replication on gemcitabine treatment 23. The present results first indicated that ORC6 was significantly upregulated in ccRCC. Interestingly, unlike ORC1-5, ORC6 could acted as an independent prognostic factor; combined with previous researches, we would imagine ORC6 may exert an irreplaceable influence on DNA replication. In our study, ORC3 expression showed no difference between normal and tumor tissue, it may play a minor role in DNA replication of ccRCC. However, further experiments are needed to verify them in ccRCC.

Elaine J. Gavin et al. first demonstrated that the expression levels of ORC6 were significantly elevated in colorectal cancer in 2008, which is consistent with the present findings in ccRCC 24. The present study observed the mRNA expression level of ORC6 was overexpressed in 72 ccRCC tumor tissues compared with in normal tissues. The expression of ORC6 was further validated in clinical ccRCC samples and cancer cells. Moreover, we found ORC6 expression was closely correlated with various cliniocopathological parameters. ORC6 may be considered a diagnostic biomarker and its expression levels tended to increase with increasing tumor stage and grade. Furthermore, the present study indicated that knockdown of ORC6 with small interfering RNA inhibited ccRCC cell wound healing ability, migration, and invasion in vitro. Moreover, ORC6 promoted cell proliferation; 96 hours after ORC6 knockdown, the proliferation rate of 786-O cells was significantly decreased. These results indicated that ORC6 serve as an oncogene, playing an important role in ccRCC.

In human colon cancer, downregulation of ORC6 sensitizes colon cancer cells to both 5-FU and cisplatin treatment. Moreover, decreased ORC6 expression in HCT-116 (wt-p53) cells induced p21 expression, which was mediated by increased level of phosphorylated p53 at ser-15 25, 26. Our GSEA results also demonstrated that the P53 signaling pathway was significantly enriched in response to high ORC6 expression in ccRCC patients.

To the best of our knowledge, the present study is the first study establishing the role of ORC6 in ccRCC tumorigenesis and progression. Our results indicated that ORC6 may be treated a potential diagnostic and prognostic biomarker for patients with ccRCC, and that ORC6 knockdown suppressed malignancy of ccRCC in vitro. However, several limitations of this study should be addressed; the specific cooperation manner of ORC6 that initiates DNA replication in ccRCC remains unsolved; the exact underlying mechanism by which ORC6 promotes ccRCC progression was not fully investigated. Therefore, we aim to explore the molecular mechanism underlying ORC6 overexpression and its associated signaling pathways in future studies.

## 5. Conclusion

In summary, our study demonstrated that ORC6 plays an oncogenic role and overexpression of ORC6 reflects poor prognosis in ccRCC. ORC6 may be a potential novel therapeutic target for ccRCC in the future.

## Supplementary Material

Supplementary tables.

## Figures and Tables

**Table 1:** Univariate and multivariate analysis of ORC6 mRNA expression and patient survival.

	Univariate analysis			Multivariate analysis^c^		
Variable	HR^a^	95%CI^b^	P	HR	95%CI	P
Overall survival						
Age (years)						
<=60 versus >60	1.759	1.287-2.406	0	1.668	1.217-2.286	0.001
Gender						
Female versus Male	0.943	0.687-1.293	0.714			
Pathological grade						
G1 or G2 versus G3 or G4	2.664	1.882-3.772	0	1.623	1.118-2.357	0.011
T stage						
T1 or T2 versus T3 or T4	3.419	2.502-4.672	0	0.945	0.515-1.737	0.856
N stage						
N0 or Nx versus N1	3.783	2.048-6.989	0	1.857	0.981-3.519	0.057
M stage						
M0 or Mx versus M1	4.39	3.196-6.029	0	2.147	1.469-3.138	0
TNM stage						
stage I or II versus stage III or IV	4.136	2.986-5.729	0	2.324	1.167-4.629	0.016
ORC6						
Low versus High	1.851	1.354-2.535	0	1.414	1.018-1.964	0.039

^a^HR estimated from Cox proportional hazard regression model; ^b^CI of the estimated HR; ^c^multivariate models were adjusted for T, N, M and G grade classification and age. CI, confidence interval; HR, hazard ratio; ORC6, origin recognition complex 6.

**Table 2:** Association between ORC6 mRNA expression and clinocopathological parameters of patients with clear cell renal cell carcinoma.

Variables		ORC6 mRNA expression			
		Low(n=265)	High(n=265)	χ^2^	P
Age (years)	≤60	120	144		
	>60	145	121	4.347	0.037
Gender	Female	90	96		
	Male	175	169	0.298	0.585
Pathological grade	G1+G2	143	106		
	G3+G4	122	159	10.37	0.001
T stage	T1+T2	193	147		
	T3+T4	72	118	17.36	<0.001
N stage	N0+Nx	262	252		
	N1	3	13	6.445	0.011
M stage	M0+Mx	239	213		
	M1	26	52	10.162	0.001
TNM stage	I+II	183	142		
	III+IV	82	123	13.732	<0.001
Survival status	Alive	201	163		
	Dead	64	102	12.666	<0.001
