# Supplementary material for: ORC6 acts as a biomarker and reflects poor outcome in clear cell renal cell carcinoma
Source: J Cancer. 2022 May 9;13(8):2504–14. doi: 10.7150/jca.71313 (PMC9174843; doi:10.7150/jca.71313)
Supplement: Supplementary file 1 — Supplementary tables. [file jcav13p2504s1.pdf]

Supplementary Table 1. Clinical characteristics of patients with ccRCC.

Characteristic	N (%)
Age	
mean ± SEM, years	54±11
Gender	
Male/female	29/21
Tumor size	
mean ± SEM, cm	4.8±3.2
Location	
Right/left	31/19
T stage	
T1a	12 (24)
T1b	14 (28)
T2a	8 (16)
T2b	6 (12)
T3	5 (10)
T4	5 (10)
N stage	
N0	42 (84)
N1	8 (16)
M stage	
M0	45 (90)
M1	5 (10)
Fuhrman grade	
1	18 (36)
2	21 (42)
3	6 (12)
4	5 (10)

SEM, standard error of the mean.

Supplementary Table 2. Univariate and multivariate analysis of ORC1 mRNA expression and patient survival.

	Univariate analysis			Multivariate analysis[c]		
Variable	HR[a]	95%CI[b]	P	HR	95%CI	P
Overall survival						
Age (years)						
<=60 versus >60	1.759	1.287-2.406	0	1.624	1.186-2.224	0.002
Gender						
Female versus Male	0.943	0.687-1.293	0.714			
Pathological grade						
G1 or G2 versus G3 or G4	2.664	1.882-3.772	0	1.67	1.152-2.420	0.007
T stage						
T1 or T2 versus T3 or T4	3.419	2.502-4.672	0	1.024	0.560-1.874	0.938
N stage						
N0 or Nx versus N1	3.783	2.048-6.989	0	1.857	0.981-3.519	0.057
M stage						
M0 or Mx versus M1	4.39	3.196-6.029	0	2.164	1.480-3.164	0
TNM stage						
stage I or II versus stage III or IV	4.136	2.986-5.729	0	2.233	1.119-4.454	0.023
ORC1						
Low versus High	1.446	1.063-1.968	0.019	1.127	0.819-1.551	0.463

[a]HR estimated from Cox proportional hazard regression model; [b]CI of the estimated HR; [c]multivariate models were adjusted for T, N, M and G grade classification and age. CI, confidence interval; HR, hazard ratio; ORC1, origin recognition complex 1.
